# Lombard’s effect’s implication in intensity, fundamental frequency and stability on the voice of individuals with Parkinson’s disease

**DOI:** 10.1016/S1808-8694(15)30129-4

**Published:** 2015-10-19

**Authors:** Araken Quedas, André de Campos Duprat, Gisele Gasparini

**Affiliations:** a M.S in Otorhinolaryngology, Otorhinolaryngologist; b PhD in Otorhinolaryngology, Professor of Otorhinolaryngology - School of Medical Sciences of Santa Casa de São Paulo; c Specialist in Voice, Speech Therapist at the Centro de Estudos da Voz. Department of Otorhinolaryngology - Medical Sciences School Santa Casa de São Paulo

**Keywords:** speech acoustics, parkinson’s disease, voice

## Abstract

Parkinson’s disease affects the central nervous system resulting in voice quality alterations. It is typically resistant to drug therapy and often persists despite extensive behavioural speech and language therapy. Previous findings show that masking noise will produce a consistent increase in voice intensity in most normal individuals (Lombard’s effect). **Aim:** we evaluated Lombard’s effect’s implication in intensity, fundamental frequency and stability on the voice of individuals with Parkinson’s disease (N=17). **Material and methods:** through acoustic analysis, we evaluated intensity alterations and fundamental frequency, before and after white masking noise 40, 70 and 90 dBSL intensities, as well as variations during each vocalization and compared with a control group (N=16). **Results:** voice intensity varied according to masking intensity, tending to non-linear increases in both groups and gender. Fundamental frequency varied, tending to non-linear increase in both groups and gender. Improvement stability occurred in fundamental frequency and vocal intensity. **Conclusion:** Lombard’s effect increased intensity, fundamental frequency and improves voice stability on these patients. Study: clinical and experimental.

## INTRODUCTION

Parkinson’s Disease (PD) was described in 1817 by an English Physician called James Parkinson, published with the title: “An assay on agitating paralysis”. It is a disease that affects the central nervous system, more specifically the neurons of the mesencephalic substantia nigra, responsible for releasing dopamine. A reduction in dopamine results in less inhibition of the basal nuclei’s activities, more precisely that of the corpus striatum, resulting in muscle rigidity, bradykinesia, tremor at rest and postural disorders^1^.

As far as speech is concerned, these patients have: intensity reduction (this is the major and most marked vocal alteration, it is progressive as the disease evolves), vocal instability, monotones, qualitative alterations such as tremor, hoarseness and pitch alterations, difficulties to start a phrase, articulatory alterations, accelerated speech and words repetition in an unconscious and uncontrolled way. This set of alterations is called hypokinetic dysarthria or disartrophonia^2^, ^3^, ^4^, ^5^, ^6^, ^7^, ^8^.

As an alternative to reduce the complaints of hypophonia in these patients, some authors have used auditory masking with the aim of improving voice intensity^11^, ^12^. This method is known as the Lombard’s Effect, which makes it natural for the individual to speak louder, because of noise exposure, preventing him to hear it properly, or because of hearing loss^9^. The mechanisms involved in this phenomenon are yet to be established^10^.

Lombard’s Effect (LE) was studied, and there was a marked improvement in voice intensity of these patients when submitted to auditory masking at 90dB SPL (decibels sound pressure level)^11^. Other authors^12^ also studied the LE’s repercussion on the voice of patients with Parkinson’s, submitting them to auditory masking with 10 and 20 dBSL (decibels sensation level), and they did not obtain the same results achieved by Adams, Lang (1992)^11^.

The little information on this topic and the lack of agreement among the few papers available, about the method or the results attained, has triggered the interest for a study assessing the behavior of sound intensity and fundamental frequency, as well as voice utterance stability in this group of patients when exposed to auditory masking.

Thus, this paper aimed at assessing, by means of an acoustic analysis, LE’s interference on the intensity and fundamental frequency in the voices of patients with PD, as well as the stability of each utterance.

## MATERIALS AND METHODS

This study was approved by the Ethics Committee of our Institution, under protocol # 070/06.

### Participants

We selected 33 participants: Patients with Parkinson’s (N=17, 8 men and 9 women) and the Control Group (N=16, 8 men and 8 women). All the participants freely signed the Informed Consent Form after being duly informed about the procedures.

### Inclusion Criteria for the Parkinson’s Group

1. Patients diagnosed with idiopathic Parkinson’s Disease (PD), without other associated neurological disorders.
2. Audiometric threshold averages equal to or below 20dB hearing level (dBHL) in the frequencies of 500, 1000 and 2000 Hz, in both ears.
3. Have vocal quality between zero and one in the item: level of dysphonia, in the GRBAS scale13.
4. Have language impairment level between zero and one in the Webster scale14 of PD disability assessment scale.
5. Have general impairment level between stages one and two in the Hoehn and Yahr’s scale15.
6. Use L-Dopa as drug treatment
7. Age between 60 and 75 years.

### Exclusion criteria for the Parkinson’s Group

We excluded those individuals who did not fit in items 1 to 7.

### Control Group

We selected 16 individuals (8 women and 8 men) without auditory or vocal complaints, with ages varying between 60 and 75 years, who were later evaluated according to items 2 and 3 as inclusion criteria for this group.

### Procedures

#### Auditory evaluation

We carried out tonal audiometry in the frequencies of 500, 1,000 and 2,000Hz in both ears.

#### Perceptive vocal assessment

An assessment, according to the GRBAS and Webster scales, carried out by three speech therapists and two otorhinolaryngologists, followed by the classification in each one of these scales, with the agreement of at least four evaluators.

#### Voice capture

Voice was captured directly to the computer by means of the Windows® sound recorder, with the microphone positioned laterally and at 5 cm from the individual’s mouth16. Each participant was submitted to uttering the vowel /a/, modal, without time control, in the following situations: without auditory masking, with auditory masking by broadband noise - “white noise”, binaural, simultaneous at 40, 70 and 90 decibels sensation level (dBSL)^17^.

#### Acoustic analysis

The data set collected was submitted to acoustic analysis by the voxmetria® software, in which we analyzed intensity variation in dB and the fundamental frequency in Hz of vocal utterance, before and after auditory masking, indirectly assessed by standard deviation within each utterance.

#### Statistical Analysis of the Data

For each parameter analyzed, we assessed the following factors: auditory masking intensity interference, the behavior of the Control and the Parkinson’s Groups, the behavior of males and females, the difference between the control and Parkinson’s groups, differences between males and females, and the behavior of gender and group factors when assessed simultaneously.

The comparisons made between Parkinson and control were expressed as average and statistically analyzed by Repetitive Measures Variance Analysis.

In all the tests, we used a significance level of 5% (p 0.05).

## RESULTS

### Intensity

**Figure 1:**
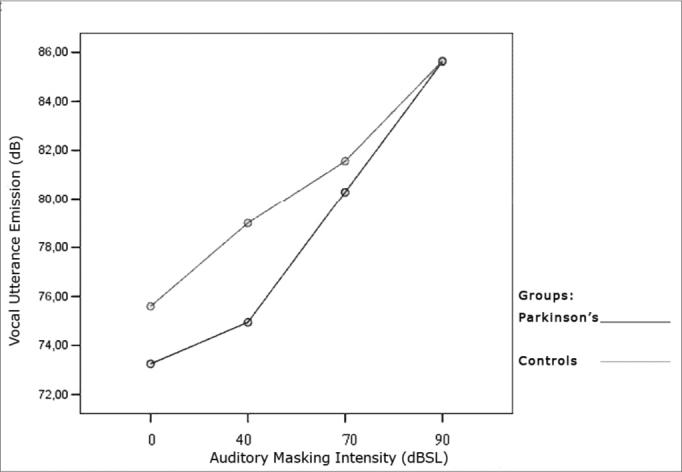
Vocal utterance intensity (dB), according to auditory masking intensity in the Control and Parkinson’s Groups.

**Figure 2:**
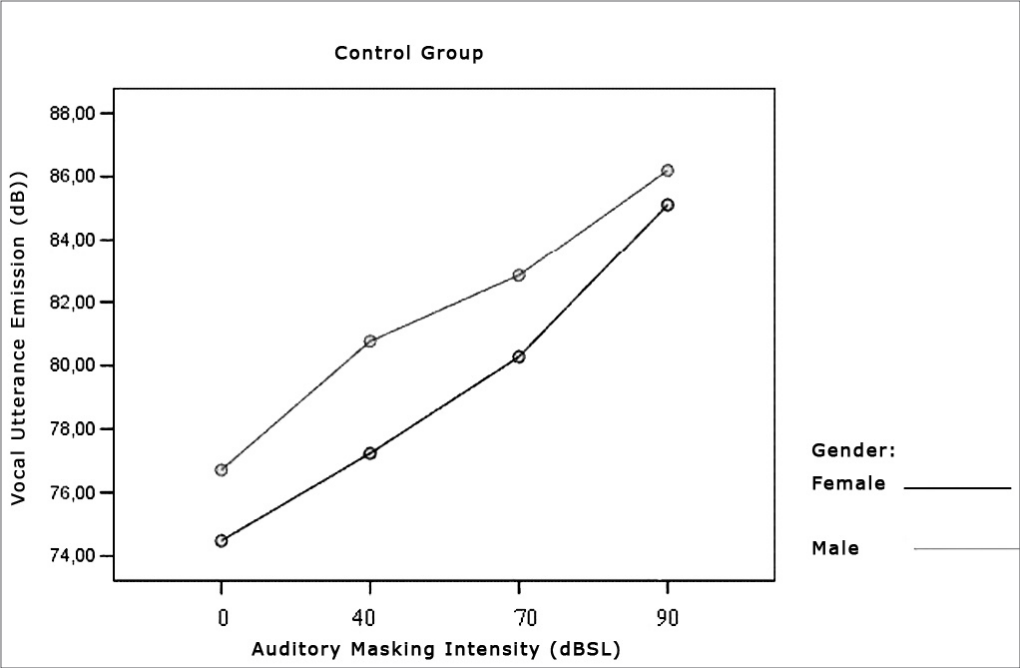
Vocal utterance intensity (dB), according to auditory masking intensity in the Control Group, males and females.

**Figure 3:**
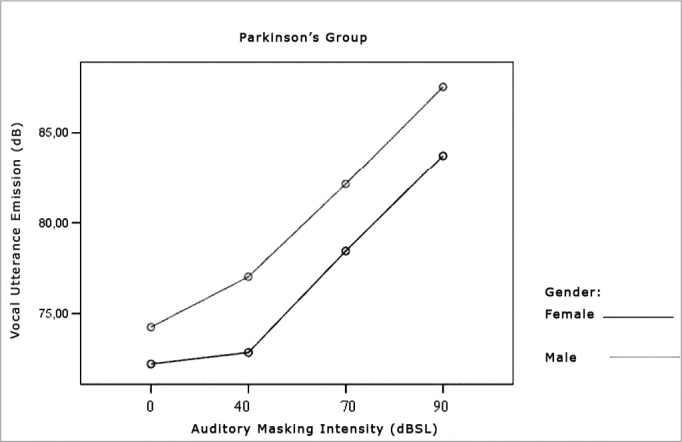
Vocal utterance intensity (dB), according to auditory masking intensity in the Parkinson’s Group, males and females.

Tables 1 and 2 show that vocal utterance intensity varies according to masking intensity (p<0.001), tending towards a non-linear increase (p<0.001). This increase is not influenced by Group (p=0.066) and nor by gender (p=0.683). There is no behavior difference between genders (p=0.240) nor between groups (p=0.430). When we compared both groups and genders simultaneously, behavior is similar (p=0.826). Such results show that, regardless of the group studied and gender, the trend is always of intensity increase. The graph represented in Figures 1, 2 and 3 show vocal utterance intensity behavior in the control and Parkinson’s Groups, as well as male and female genders.

**Table 1:** Utterance intensity average in dB, according to the auditory masking, by group and gender.

Masking	Utterance intensities mean values (dB)					
	Control Group			Parkinson’s Group		
	Male	Female	Total	Male	Female	Total
0dB	76,7	74,5	75,6	74,3	72,2	73,2
40dB	79,0	75,0	77,1	77,0	72,8	74,9
70dB	82,8	80,3	81,6	82,1	78,4	80,3
90dB	86,2	85,1	85,7	87,5	83,7	85,6

**Table 2:** Vocal utterance intensity repetitive measures variance analysis.

Factor	p value
Masking intensity	< 0.001
Group interaction	0.066
Gender interaction	0.683
Group differences	0.430
Gender differences	0.240
Group x gender interaction	0.826

### Intensity stability within each vocal utterance

This parameter was indirectly assessed through the intensity standard deviation within each vocal utterance.

**Figure 4:**
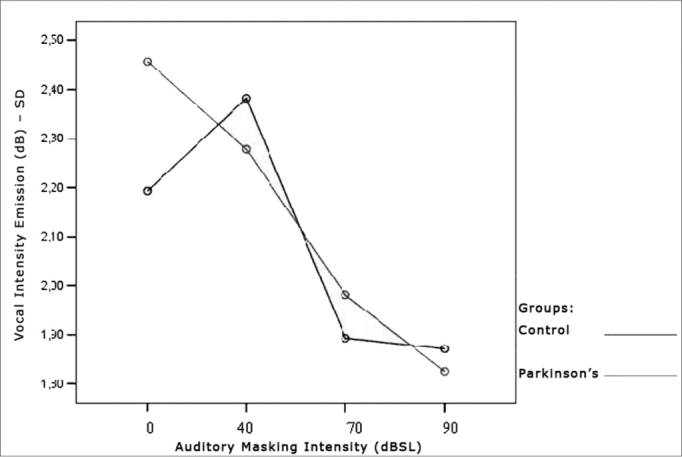
Vocal utterance intensity standard deviation (dB), according to auditory masking intensity, Control and Parkinson’s Group.

**Figure 5:**
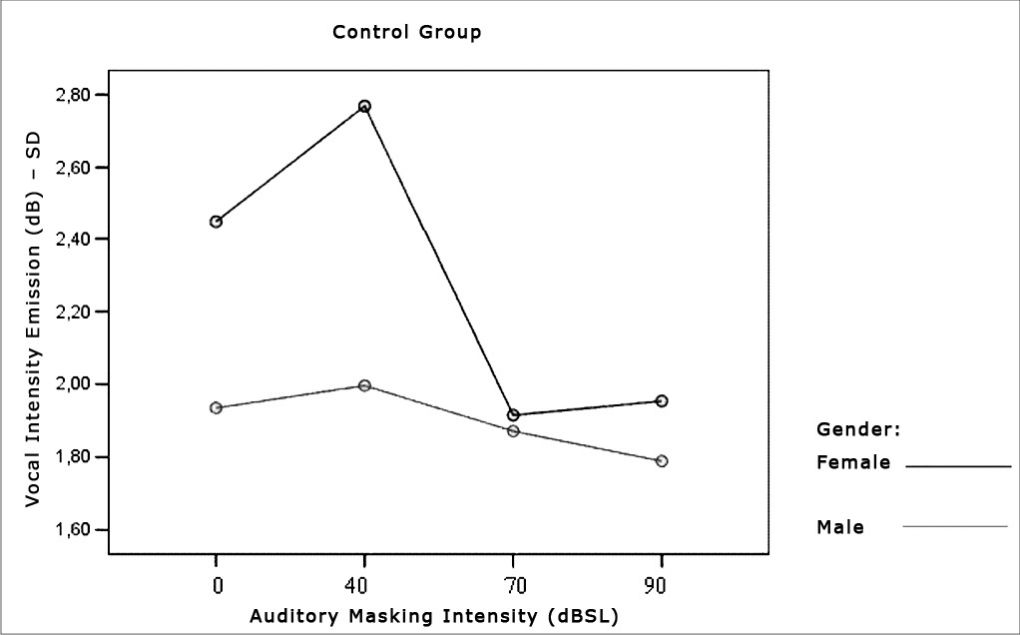
Vocal utterance intensity standard deviation (dB), according to auditory masking intensity, Control group, males and females.

**Figure 6:**
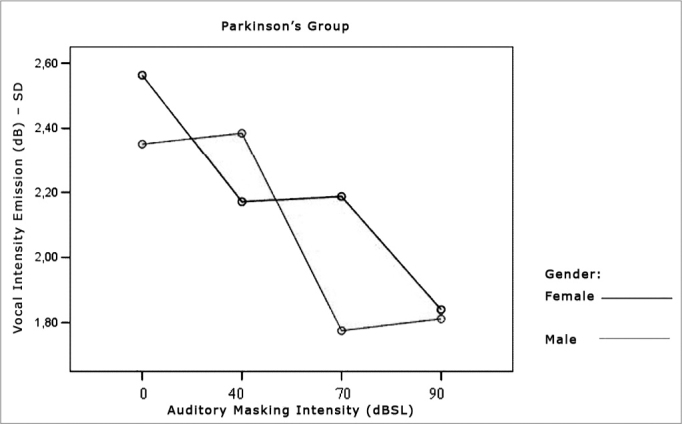
Vocal utterance intensity standard deviation (dB), according to auditory masking intensity, Parkinson’s Group males and females.

Tables 3 and 4 show that the intensity standard deviation within each vocal utterance varies according to masking intensity (p<0.001), tending towards a non-linear reduction (p<0.001). This reduction is not influenced by group (p=0.557), nor by gender (p=0.807). There is no behavior difference between the genders (p=0.180) nor between the groups (p=0.776). When compared both groups and genders simultaneously, behavior was similar (p=0.460). Thus, the results show a trend towards stability, with a more uniform vocal utterance as far as intensity is concerned. The graphs shown on Figures 4, 5 and 6 present the intensity standard deviation behavior in each utterance in the Control and Parkinson’s Groups, as well as in males and females.

**Table 3:** Mean values of the intensity standard deviations within each vocal utterance in dB, according to auditory masking by group and gender.

Masking	Utterance intensities standard deviation mean values (dB)					
	Control Group			Parkinson’s Group		
	Male	Female	Total	Male	Female	Total
0dB	2,4	1,9	2,1	2,6	2,3	2,5
40dB	2,7	2,0	2,4	2,2	2,4	2,3
70dB	1,9	1,8	1,9	2,2	1,8	2,0
90dB	1,9	1,8	1,9	1,8	1,8	1,8

**Table 4:** Repetitive measures variance analysis for the intensity standard deviation within each vocal utterance.

Factor	p Value
Masking intensity	< 0.001
Group interaction	0.557
Gender interaction	0.807
Group differences	0.776
Gender differences	0.180
Group x gender interaction	0.460

### Vocal utterance frequency

**Figure 7:**
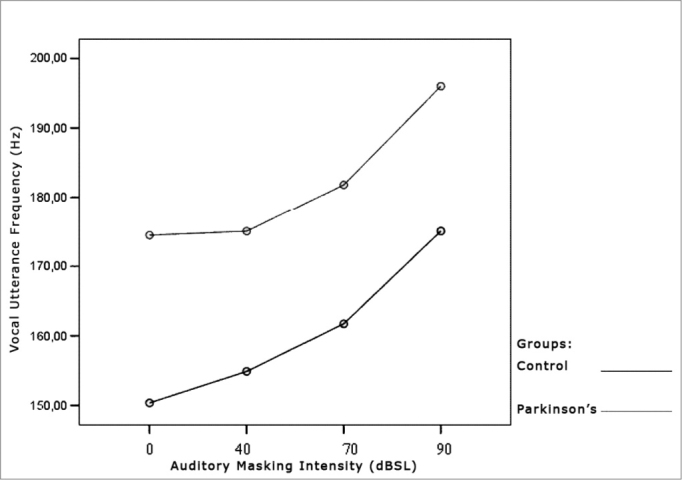
Vocal utterance frequency (Hz), according to auditory masking intensity, Control and Parkinson’s groups.

**Figure 8:**
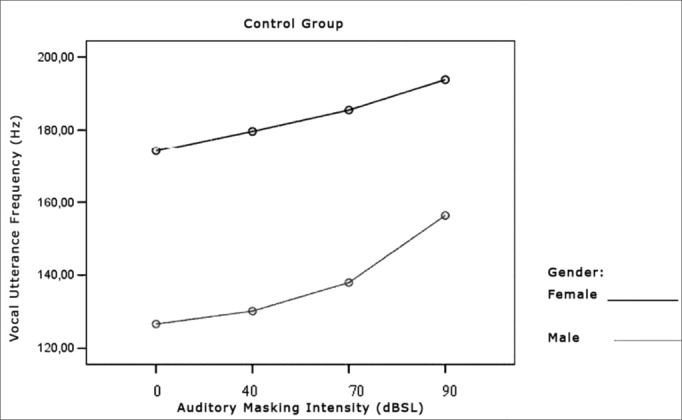
Vocal utterance frequency (Hz), according to auditory masking intensity, Control group, males and females.

**Figure 9:**
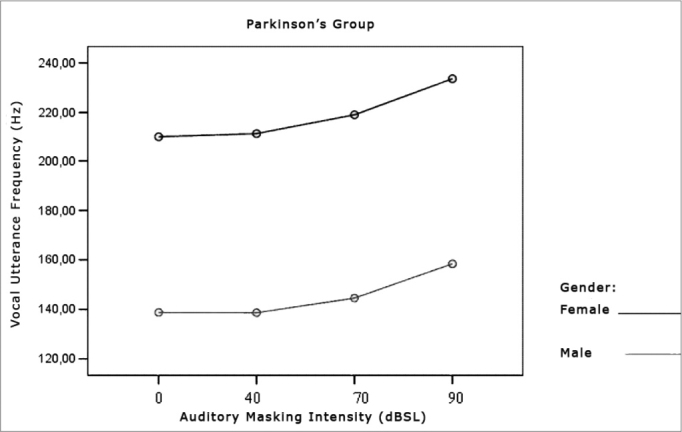
Vocal utterance frequency (Hz), according to auditory masking intensity, Parkinson’s group, males and females.

Tables 5 and 6 show that vocal utterance frequency varies according to masking intensity (p<0.001), tending towards non-linear increase (p<0.001). This increase is not influenced by group (p=0.747) nor by gender (p=0.640). There is a behavior difference between genders (p<0.001), and it was higher for females. Repetitive measures variance analysis suggests a difference between groups (p=0.056), which was higher in the Parkinson’s group. When we compared both groups and gender simultaneously, the behavior was similar (p=0.201). This result means that, in both groups and genders there is an increase in vocal utterance frequency during exposure to auditory masking. The behavior in both the Control and Parkinson’s groups, as well as in males and females can be seen in the charts shown in Figures 7, 8 and 9.

**Table 5:** Utterance frequencies average in Hz, according to auditory masking, by group and gender.

Masking	Utterance frequencies average (Hz)					
	Control Group			Parkinson’s Group		
	Male	Female	Total	Male	Female	Total
0dB	126,6	174,1	150,4	138,7	210,2	172,4
40dB	130,1	179,6	154,9	138,6	211,4	172,9
70dB	137,9	185,5	161,7	144,6	219,1	179,6
90dB	156,3	193,8	175,1	158,4	233,6	193,8

**Table 6:** Variance analysis of the repeated vocal utterance frequency.

Factor	p Value
Masking intensity	< 0.001
Group interaction	0.747
Gender interaction	0.640
Group differences	0.056
Gender differences	< 0.001
Group x gender interaction	0.201

### Frequency stability within a given vocal utterance

This parameter was assessed indirectly through the frequency standard deviation within each vocal utterance.

**Figure 10:**
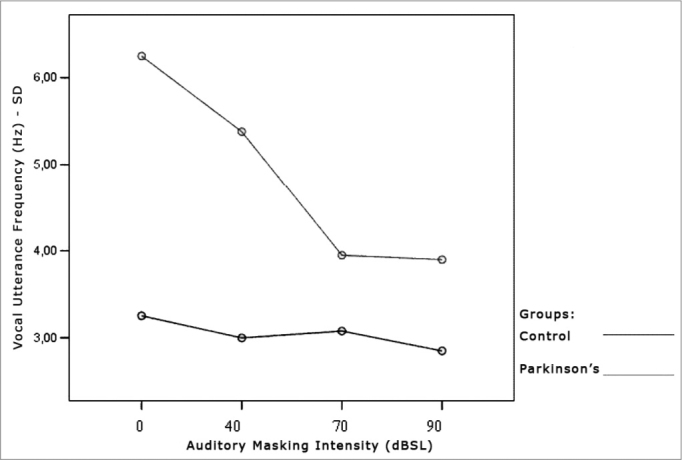
Vocal utterance frequency standard deviation (Hz), according to auditory masking intensity, Control and Parkinson’s groups.

**Figure 11:**
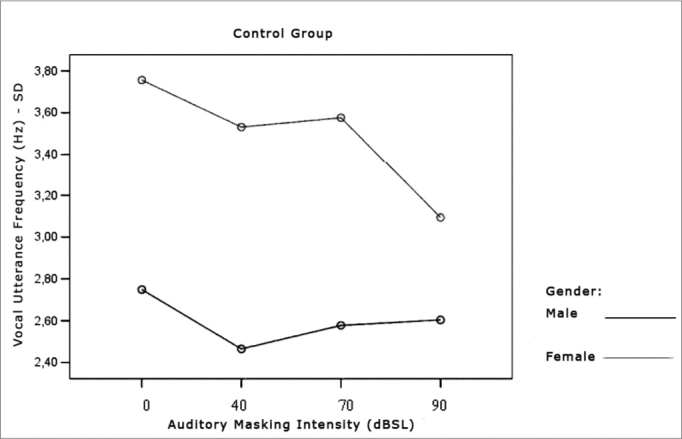
Vocal utterance frequency standard deviation (Hz), according to auditory masking intensity, Control group, males and females.

**Figure 12:**
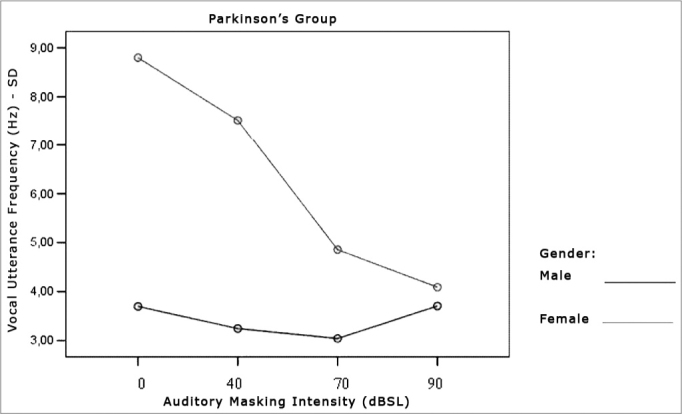
Vocal utterance frequency standard deviation (Hz), according to auditory masking intensity, Parkinson’s group, males and females.

Tables 7 and 8 show that the frequency standard deviation within each vocal utterance varies according to masking intensity (p<0.001), tending towards non-linear reduction (p<0.001). This reduction occurs differently in the different groups (p=0.012) and genders (p=0.002). This behavior difference does not allow testing the differences between the Control and the Parkinson’s Group and between males and females. It is possible to suggest that there is a trend towards vocal utterance frequency stability. The graphs presented in Figures 10, 11 and 12 show different behavior between the Control and Parkinson’s groups, as it happens between males and females in both groups.

**Table 7:** Average value of frequency standard deviation within each vocal utterance in Hz, according to auditory masking, by group and gender.

Masking	Mean values of the standard deviation in the utterance frequencies (Hz)					
	Control Group			Parkinson’s Group		
	Male	Female	Total	Male	Female	Total
0dB	2,7	3,7	3,2	3,7	8,8	6,1
40dB	2,5	3,5	3,0	3,2	7,5	5,2
70dB	2,6	3,6	3,1	3,0	4,8	3,9
90dB	2,6	3,1	2,8	3,7	4,1	3,9

**Table 8:** Variance analysis of the frequency standard deviation measures within each vocal utterance.

Factor	p Value
Masking intensity	<0.001
Group interaction	0.012
Gender interaction	0.002
Group difference	0.003
Gender difference	0.002
Group x gender difference	0.086

## DISCUSSION

Voice neural control involves integration between the somatic motor system and the limbic system, involving fibers that go from the cortex, passing through the basal nuclei and by the mesencephalic substantia nigra^18^. The basal nucleus plays an important role in modulating cortical impulses^19^. Such modulation is reduced in PD, because of degeneration in the substantia nigra present at the base of the mesencephalus, involving not only dopaminergic neurons, but also other structures that produce serotonin, noradrenalin and acetylcholine in the genesis of the disease. The affected zone influences extra-pyramidal motor control, in other words, they control autonomous movements such as facial muscles (unconscious emotional communication), for example. Moreover, these neurons modify basic conscious commands coming from motor cortical neurons in a way to execute movements smoothly and without losing balance. It is also this extrapyramidal system that prevents continuous contraction or relaxation, and these events directly affect voice production^20^, ^21^.

Thus, voice and speech difference, known as dysarthrophonia, represent an important set of signs and symptoms in PD. Traditional methods of phonotherapeutic treatment were not efficient to treat these alterations^6^. Currently, LSVT® has been the most used technique, and the one showing the best results^6^, ^21^. Some authors have proposed some alternatives to improve these patients^11^, ^12^, ^22^, however, the only statistically proven method is LSVT®^21^.

This limited number of alternatives was the major driver for this study, and besides, the method proposed is simple to use and it can be improved in the future to be used in screening patients for treatment, indicating, for instance, those patients with better prognoses.

### Discussion of the results

In order to facilitate results discussion, each parameter was analyzed separately.

### Vocal utterance intensity

Utterance intensity increased in both groups evaluated and in both genders, matching results found in the literature^9^, ^11^, ^23^, ^24^, ^25^. This increase was progressive and proportional to the increase in masking that is offered, that is, the greater the masking, the greater will be vocal utterance intensity, in agreement with studies previously published (Figure 1, Figure 2, Figure 3)^9^, ^11^, ^12^, ^26^, ^27^, ^28^, ^29^, ^30^.

However, the initial average intensity of auditory pre-masking utterance was reduced in the group with Parkinson’s when compared to the Control group (Figure 1), confirming hypophonia as an important characteristic in these individuals^3^, ^5^, ^6^, ^11^, ^12^, ^31^.

All the individuals in the Parkinson’s group used levodopa or dopaminergic agonists as a pharmacologic measure. There is no consensus in the literature as to voice improvements with the use of levodopa and dopaminergic agonists. Some papers suggest that pharmacologic treatment does not bring about significant improvements in vocal traits^31^, ^32^. Others suggest that speech improvements due to medication use occur mainly with the symptoms related with joint and posture, considered dopamine-dependents, and were not very much felt in parameters such as tremor^5^, ^33^. There are those that advocate that drug treatment promotes significant speech improvements^34^, ^35^. In our study, although there has been an increase in voice intensity, both in auditory perception and through acoustic analysis, utterance intensity in the Parkinson’s group was less, suggesting that the “pharmacological treatment” factor, although bringing some voice improvement, is not enough to normalize patterns.

Results suggest that, as it happens with vocal therapy, the goal in voice intensity increase is reached through LE^6^, ^11^, ^12^, ^21^, ^22^, ^36^.

### Vocal Utterance Intensity Instability

This parameter was indirectly checked by means of a standard deviation pattern within each vocal utterance. It tended towards reduction in both groups, varying proportionally in relation to masking intensity. That is, the more intense the masking, the lower is the vocal utterance standard deviation. There was no behavior difference between Parkinson’s and the Control group. It is possible to visually perceive these findings (Figure 4).

Such findings suggest that an increase in vocal utterance intensity improves stability in terms of intensity, and this improvement was similar in both groups. This is an important finding in patients with PD, since one of the characteristics of this group is the difficulty in maintaining vocal utterance stability during speech^4^, ^5^.

There are no papers in the literature describing the use of this analysis parameter. Thus, the comments hereby made were based on acoustic and perception analysis findings, and on statistical analysis of the data.

### Vocal Utterance Frequency

Utterance frequency increased in both groups assessed and in both genders. This increase was progressive and proportional to the increase in masking used, that is, the higher the masking, the greater the vocal utterance intensity reflecting in a higher fundamental frequency and a greater vocal utterance power^37^. Average utterance frequency was greater in females, which did not confirm the findings from Holmes et al. (2000)^5^, who found a reduction in the fundamental frequency in women and an increase in men. Perception and auditory findings match the results from the acoustic analysis, also in disagreement from the findings by Holmes et al. (2000)^5^. A hypothesis that could explain this disagreement is the fact that patients in this study were in the initial stages of the disease, while those from the study aforementioned belonged to many stages of the disease.

As to the fundamental frequency in these patients, literature is scarce, showing that their major interest is utterance intensity, followed by the alterations that mark the instability. In this sense, it is important to assess vocal utterance frequency in PD patients.

### Vocal Utterance Frequency Stability

Just as it happened with vocal utterance intensity stability, this parameter was indirectly assessed by means of the fundamental frequency standard deviation within each vocal utterance. In both groups, this parameter tended to reduce, varying proportionally in relation to masking intensity. That is, the more intense the masking, the lower is vocal utterance frequency standard deviation. However, the behavior difference did not let us test differences between the Control and the Parkinson’s Groups as far as gender is concerned. It is possible to suggest that there is a trend towards vocal utterance frequency stability.

The comments made hereby were based on evaluating the Graph (Figure 10) that represent the analysis of the parameter tested, assessing the behavior of both, the Control and the Parkinson’s Groups.

The Graph (Figure 10) shows that the control group curve tends to be flatter, suggesting greater frequency stability in vocal utterance in this group. In the Parkinson’s group, values start high and tend to stabilize after masking with 70dBSL. This stability is important for total voice improvement in these patients^4^, ^5^.

Literature has no paper describing the use of such analysis parameter. The comments hereby made were based on the findings of acoustic analyses, perception and auditory analysis, and data statistical analysis.

## FINAL REMARKS

If we consider that speech mirrors our personalities, which is unique in its vibrations, tones and musicality, it becomes easier to understand how much these changes interfere in the daily activities of these patients and in their social relations. However, the means specifically created for language improvement in these patients are still limited, currently being restricted to LSVT®. Surgical treatment is still far from us. The “Deep Brain Stimulation”, whose goal is to reduce the activities of the globus pallidus, subthalamic nucleus and thalamus, is a great promise for these patients^38^. Postoperative results have been very promising with important voice improvements, showing the way to a new therapeutic horizon^39^, ^40^, ^41^, ^42^. However, we have to take into account that this technology is not available to most of these patients, especially in a developing country like Brazil, thus the importance of developing innovative and accessible approaches that help in assessing and treating vocal symptoms in this group. Studies such as those from Adams, Lang (1992)^11^, and Angelis et al. (1997)^22^ and Ho et al. (1999)^12^ are highly important because, besides opening new therapeutic horizons, have fostered new studies in search of a better quality of life for this group.

## CONCLUSION

The Lombard effect causes significant increase in vocal utterance fundamental frequency for individuals with Parkinson’s disease. Results also suggest an improvement in vocal utterance stability, both in terms of intensity and fundamental frequency. When we compare the results between the Control and the Parkinson’s Group, both present similar behavior, suggesting that the LE occur in both groups apparently in the same way.

## References

[1] de Mattos JP, Meneses MS, Teive HAG (1996). Doença de Parkinson. Aspectos clínicos e cirúrgicos.

[2] Logemann JA, Fisher HB, Boshes B, Blonsky ER (1978). Frequency and occurrence of vocal tract dysfunctions in the speech of a large sample of Parkinson patients. J Speech Hear Disord.

[3] Streifler M, Hofman S (1984). Disorders of verbal expression in parkinsonism. Adv Neurol.

[4] Illes J, Metter EJ, Hanson WR, Iritani S (1988). Language production in Parkinsons disease: acoustic and linguistic considerations. Brain Lang.

[5] Holmes RJ, Oates JM, Phyland DJ, Hughes AJ (2000). Voice characteristics in the progression of Parkinsons disease. Int J Lang Commun Disord.

[6] Dias AE, Limongi JCP (2003). Treatment of vocal symptoms in Parkinsons disease: the Lee Silverman method. Arq Neuropsiquiatr.

[7] Blumin JH, Pcolinsky DE, Atkins JP (2004). Laryngeal findings in advanced Parkinsons disease. Ann Otol Rhinol Laryngol.

[8] Rosen KM, Kent RD, Duffy JR (2005). Task-based profile of vocal intensity decline in Parkinsons disease. Folia Phoniatr Logop.

[9] Lombard E (1911). Le signe de lelevation de la voix. Ann maladie oreille larynx nez pharynx.

[10] Nonaka S, Takahashi R, Enomoto K, Katada A, Unno T (1997). Lombard reflex during PAG-induced vocalization in decerebrate cats. Neurosci Res.

[11] Adams SG, Lang A (1992). E. Can the Lombard effect be used to improve low voice intensity in Parkinsons disease?. Eur J Disord Commun.

[12] Ho AK, Bradshaw JL, Iansek R, Alfredson R (1999). Speech volume regulation in Parkinsons disease: effects of implicit cues and explicit instructions. Neuropsychologia.

[17] Almeida K, Russo ICP, Momensohn-Santos TM (2001). A aplicação do mascaramento em audiologia.

[18] Fernandes AMF (1999). Estudo dos aspectos neurológicos do Controle Motor Laríngeo [dissertação-mestrado].

[19] Gerfen CR (1992). The Neoestriatal Mosaic: Multiple levels of compartmental organization in the basal ganglia. Annu Ver Neurosci.

[20] Mink JW (2003). The basal ganglia and involuntary movements, impaired inhibition of competing motor patterns. Arch Neurol.

[21] Ramig LO, Countryman S, OBrien C, Hoehn M, Thompson L (1996). Intensive speech treatment for patients with Parkinsons disease: short-and long-term comparison of two techniques. Neurology.

[22] de Angelis EC, Mourao LF, Ferraz HB, Behlau MS, Pontes PA, Andrade LA (1997). Effect of voice rehabilitation on oral communication of Parkinsons disease patients. Acta Neurol Scand.

[23] Siegel GM, Schork EJ, Pick HL, Garber SR (1982). Parameters of auditory feedback. J Speech Hear Res.

[24] Amazi DK, Garber SR (1982). The Lombard sign as a function of age and task. Speech Hear Res.

[25] Zeine L, Brandt JF (1988). The Lombard effect on alaryngeal speech. J Commun Disord.

[26] Black JW (1951). The effect of delayed side-tone upon vocal rate and intensity. J Speech Disord.

[27] Black JW (1955). The persistence of the effects of delayed side-tone. J Speech Disord.

[28] Lane H, Tranel B (1971). The Lombard sign and the role of hearing in speech. J Speech Hear Res.

[29] Letowski T, Frank T, Caravella J (1993). Acoustical properties of speech produced in noise presented through supra-aural earphones. Ear Hear.

[30] Winkworth AL, Davis PJ (1997). Speech breathing and the Lombard effect. J Speech Lang Hear Res.

[31] Gamboa J, Jimenez-Jimenez FJ, Nieto A, Montojo J, Orti-Pareja M, Molina JA, Garcia-Albea E, Cobeta I (1997). Acoustic voice analysis in patients with Parkinsons disease treated with dopaminergic drugs. J Voice.

[32] Stewart C, Winfield L, Hunt A, Bressman SB, Fahn S, Blitzer A, Brin MF (1995). Speech dysfunction in early Parkinsons disease. Mov Disord.

[33] Goberman AM (2005). Correlation between acoustic speech characteristics and non-speech motor performance in Parkinson Disease. Med Sci Monit.

[34] Azevedo LL, Cardoso F, Reis C (2000). Acoustic analysis of prosody in females with Parkinsons disease: effect of L-dopa. Arq Neuropsiquiatr.

[35] Azevedo LL, Cardoso F, Reis C (2003). Acoustic analysis of prosody in females with Parkinsons disease: comparison with normal controls. Arq Neuropsiquiatr.

[36] Ramig LO, Verdolini K (1998). Treatment efficacy: voice disorders. J Speech Lang Hear Res.

[37] Hsiao TY, Solomon NP, Luschei ES, Titze IR, Liu K, Fu TC, Hsu MM (1994). Effect of subglottic pressure on fundamental frequency of the canine larynx with active muscle tensions. Ann Otol Rhinol Laryngol.

[38] Panikar D, Kishore A (2003 Jun). Deep brain stimulation for Parkinsons disease. Neurol India.

[39] Schulz GM, Peterson T, Sapienza CM, Greer M, Friedman W (1999). Voice and speech characteristics of persons with Parkinsons disease pre- and post pallidotomy surgery: preliminary findings. J Speech Lang Hear Res.

[40] Schulz GM, Grant MK (2000). Effects of speech therapy and pharmacologic and surgical treatments on voice and speech in Parkinsons disease: a review of the literature. J Commun Disord.

[41] Nagulic M, Davidovic J, Nagulic I (2005). Parkinsonian voice acoustic analysis in real-time after stereotactic thalamotomy. Stereotact Funct Neurosurg.

[42] Mourão LF, Aguiar PM, Ferraz FA, Behlau MS, Ferraz HB (2005). Acoustic voice assessment in Parkinsons disease patients submitted to posteroventral pallidotomy. Arq Neuropsquiatr.

